# Malaria control among Myanmar migrants in Thailand: a qualitative study of healthcare providers

**DOI:** 10.1186/s12936-025-05397-4

**Published:** 2025-05-22

**Authors:** Nichakan Inthitanon, Piyarat Sripoorote, Yupaporn Wattanagoon, Pattamaporn Petchvijit, Ammarind Anantjitsupha, Kyawt Mon Win, Nattawan Rachaphaew, Khaing Zin Zin Htwe, Kritsana Suk-aum, Peeriya Watakulsin, Liwang Cui, Jetsumon Sattabongkot, Daniel M. Parker, Wang Nguitragool, Pyae Linn Aung

**Affiliations:** 1 Mahidol Vivax Research Unit, Faculty of Tropical Medicine, Mahidol University, Bangkok, Thailand; 2 Department of Clinical Tropical Medicine, Faculty of Tropical Medicine, Mahidol University, Bangkok, Thailand; 3 Center of Vector Borne Disease Control 2.3, Ministry of Public Health, Nonthaburi, Thailand; 4 Office of Disease Prevention and Control 2, Ministry of Public Health, Nonthaburi, Thailand; 5 Division of Infectious Diseases and International Medicine, Department of Internal Medicine, Morsani College of Medicine, University of South Florida, 3720 Spectrum Boulevard, Suite 304, Tampa, FL 33612 USA; 6 Department of Population Health and Disease Prevention, Department of Epidemiology, University of California, Irvine, CA USA; 7 Department of Molecular Tropical Medicine and Genetics, Faculty of Tropical Medicine, Mahidol University, Bangkok, Thailand

**Keywords:** Malaria, Migrants, Myanmar, Thailand, Border, Surveillance, Challenges

## Abstract

**Background:**

Thailand has experienced a recent surge in malaria cases, particularly along the Thailand-Myanmar border, likely driven by the importation of infections by Myanmar migrants. Implementing malaria control measures, especially surveillance among these high-risk populations, presents significant challenges. This study aimed to identify key obstacles and propose targeted solutions for enhancing malaria control among Myanmar migrants in border areas of Thailand.

**Methods:**

A cross-sectional qualitative study was conducted in early 2024. Semi-structured interviews were held with 20 government healthcare providers and village health volunteers involved in malaria control across three districts in western Thailand with the highest malaria caseloads. Data were analysed using thematic analysis.

**Results:**

Respondents consistently linked the rise in malaria cases to increased cross-border migration from Myanmar following recent political unrest. Key challenges included difficulty locating and following up with short-term or undocumented migrants, language barriers that hinder health education, and delays in diagnosis because of limited infrastructure and reliance on rapid diagnostic tests in areas without electricity. Suggested solutions included deploying mobile malaria posts near informal border crossings, mandating malaria testing before worksite entry, and engaging local employers and community leaders to register new arrivals and support treatment adherence among migrants.

**Conclusions:**

Ongoing political instability in Myanmar continues to drive a large and dynamic population of migrants into Thailand’s border regions, sustaining malaria importation and complicating elimination efforts. Tailored, migrant-responsive strategies, such as mobile surveillance near border crossings, community-based follow-up mechanisms, and infrastructure improvements, are urgently needed to close coverage gaps and achieve malaria elimination by 2030.

## Background

The Greater Mekong Subregion (GMS) consists of six countries with shared land borders where cross-border population mobility plays a critical role in malaria transmission dynamics [1, 2]. Among these countries, China achieved malaria elimination by June 2021 [3]. Other countries, such as Cambodia, Laos, and Vietnam, have made significant progress in malaria control, with residual transmission confined to specific regions [4–7]. Although malaria cases in Thailand steadily declined from 2016 to 2021, a resurgence occurred in 2023, with approximately 17,000 reported cases—a sevenfold increase compared to 2021 and twice the number reported in 2022 [4, 8]. Notably, around 45% of these cases involved Thai residents, while the remaining cases were among short- and long-term migrants, primarily from Myanmar [8]. In contrast, Myanmar continues to experience a persistent malaria burden, with a recent surge in cases following the February 2021 military coup, which has severely weakened the healthcare system [4]. This situation has led to increased population displacement as people seek work opportunities and means of survival, primarily along border areas such as the Thailand-Myanmar border [9].

In Thailand, malaria surveillance activities in endemic areas are typically conducted by healthcare staff at malaria health facilities, including malaria clinics and posts, supported by trained village health volunteers (VHVs) under the guidance of the Department of Disease Control, Ministry of Public Health [10, 11]. Since 2016, Thailand has implemented the “1–3–7” strategy, adapted from China, aimed at interrupting onward malaria transmission from index cases [12, 13]. Briefly, the strategy involves notifying cases within one day, conducting case investigations within three days, and performing reactive case detection (RCD) within seven days. While this approach has shown promise, a study conducted in Thailand found that by 2021, 88% of cases were notified within 24 h, 96% underwent case investigation within three days, and 80% of RCD activities were completed within seven days [13]. The success and sustainability of the 1–3–7 strategy depend on various factors, including sufficient labour force, sustained funding, adequate supplies, high-quality case management, and accurate surveillance data [12, 14]. Similar challenges were reported during the early phase of 1–3–7 implementation in Myanmar, where difficulties included inadequate supplies, transportation issues, delays in reporting, discrepancies in data, and an influx of internally displaced people [15].

Despite Thailand's ongoing efforts to control the rising number of malaria cases, particularly in border regions, several challenges remain, including the emergence of multidrug resistance, biological diversity among malaria mosquito vectors, ethnic and linguistic diversity, and a complex socio-political context [16–18]. Effective vector control is hampered by the remote habitats of malaria vectors. For instance, Tak Province, which has the highest malaria burden, is home to major malaria vectors, such as *Anopheles dirus*, *Anopheles minimus*, and *Anopheles maculatus*, along with emerging insecticide resistance [19–21]. Additionally, as a border region, it experiences a high influx of Myanmar migrants, who are often vulnerable to malaria because of exposure to malaria vectors which is likely facilitated by poor access to preventive measures, real or perceived barriers to diagnosis and treatment when ill, and poor living conditions [22, 23].

Non-citizens residing in Thailand (including displaced individuals) are classified as either short-term (M2) or long-term (M1) migrants, based on whether their stay is less than or greater than six months. As of 2023, estimates suggest that approximately four million migrants reside in Thailand, with about 75% originating from Myanmar [9]. Although Thailand provides free malaria diagnosis, treatment, and preventive tools, including long-lasting insecticidal nets (LLINs), to all individuals regardless of their migration status, Myanmar migrants often face barriers to accessing malaria services. These barriers include language difficulties, low education levels, unfamiliarity with the Thai healthcare system, and concerns related to migration status and associated healthcare costs [22, 24, 25]. Studies have reported a higher prevalence of malaria among Myanmar migrants and lower usage of preventive measures such as LLINs compared to Thai residents [23, 26]. These factors create significant gaps between the provision of malaria services and the reach to migrant populations.

The Thailand–Myanmar border is notably porous, with numerous informal crossing points that complicate malaria surveillance and case management among those who traverse the border. Untracked cross-border movements hinder timely diagnosis, treatment completion, especially for *Plasmodium vivax* infections, and facilitate ongoing transmission. To support Thailand’s goal of malaria elimination by 2030, it is critical to understand and to subsequently address the barriers in delivering malaria services to at-risk mobile populations. The present study aims to explore the challenges faced by healthcare providers in delivering malaria control services to Myanmar migrants and to identify potential solutions. The findings will help inform the development of evidence-based, tailored interventions targeting these at-risk populations, ultimately contributing to the goal of malaria elimination in Thailand.

## Methods

### Study design

This study employed a cross-sectional design conducted in February 2024. Qualitative data were collected from healthcare providers involved in malaria control across three districts along the Thailand-Myanmar border.

### Study locations

**Fig. 1:**
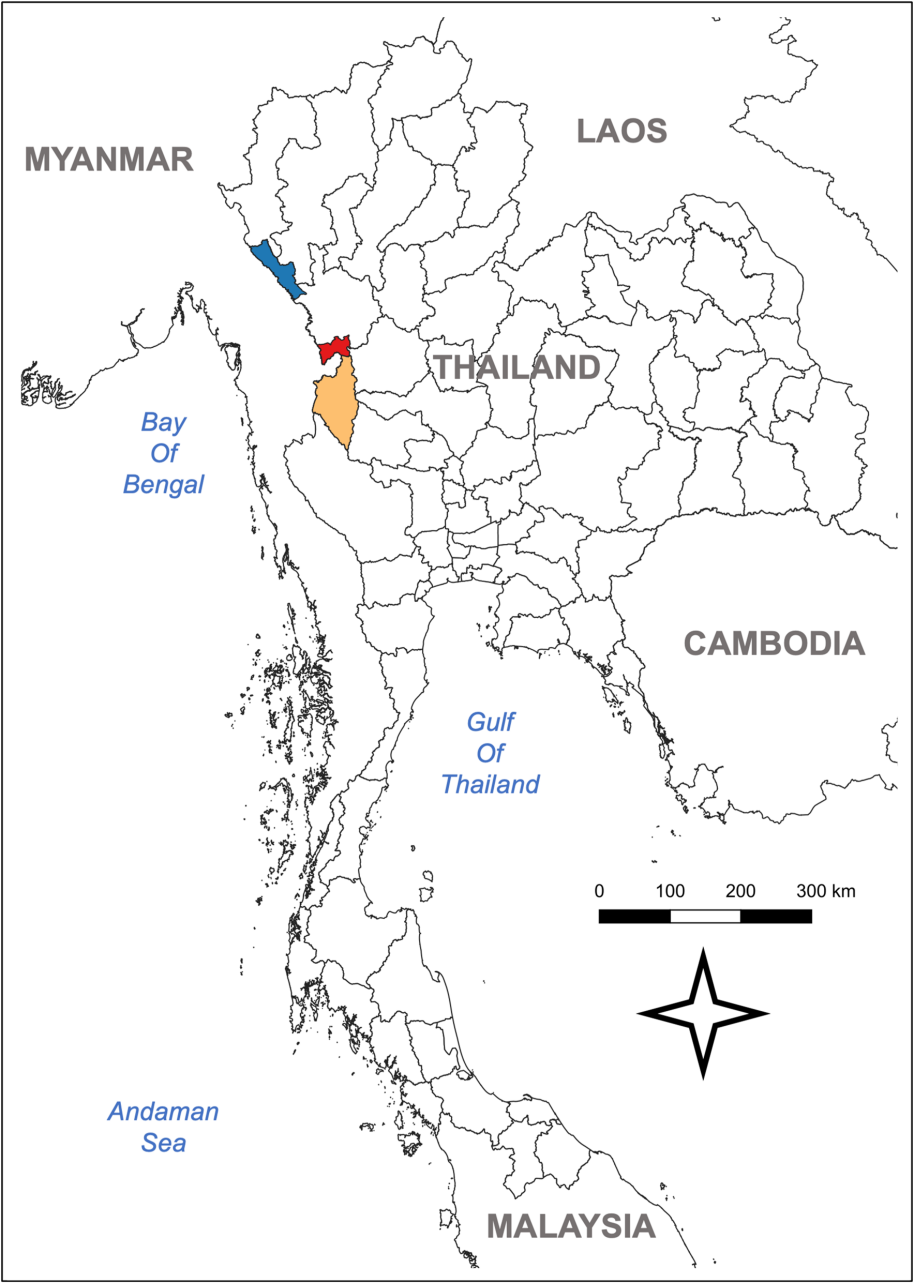
Map showing the three study areas in western Thailand

**Fig. 2:**
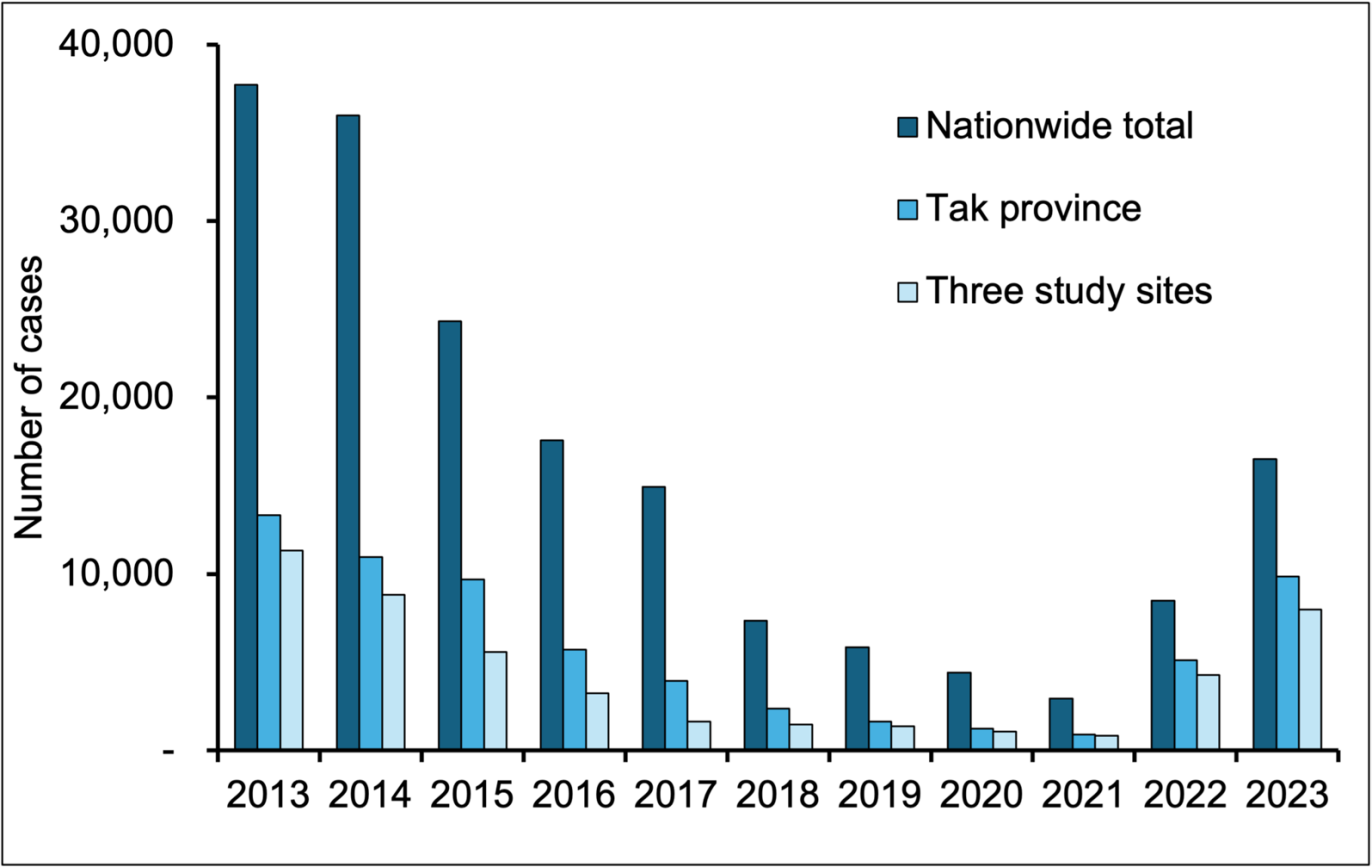
Malaria trends in Thailand, Tak province, and the three selected study sites (2013–2023)

Among Thailand’s 76 provinces, Tak province, located in the western region, was purposively selected as the study area due to its high malaria burden in 2022 and 2023. In 2023, approximately 10,000 malaria cases were reported in Tak, accounting for around 60% of the country’s total malaria cases. Notably, only 38% of these cases involved Thai residents, indicating a significant malaria problem among migrants, particularly those from Myanmar [8]. Out of the nine districts in Tak, three with the highest number of malaria cases were selected for this study (Fig. 1). These three districts accounted for over 80% of the total malaria cases in Tak and more than 48% of total nationally reported cases in 2023 (Fig. 2).

### Study samples and recruitment

Thailand’s malaria healthcare system consists of multiple service points, including hospitals, malaria clinics, malaria posts, and VHVs, where individuals can receive diagnosis and treatment for malaria. Within this system, Thailand operates a semi-vertical structure for malaria control and elimination efforts, wherein specialized malaria services, such as surveillance, diagnosis, treatment, and follow-up, are managed through dedicated malaria units operating in parallel with the general health services. For this study, data were collected from various components of the malaria control system, primarily following the vertical reporting line overseen by the Division of Vector-Borne Diseases. Hospital settings were excluded, as they operate under the general healthcare system managed by the Bureau of Epidemiology, while VHVs were included to better represent community-level malaria control efforts, particularly those targeting migrant populations.

**Fig. 3:**
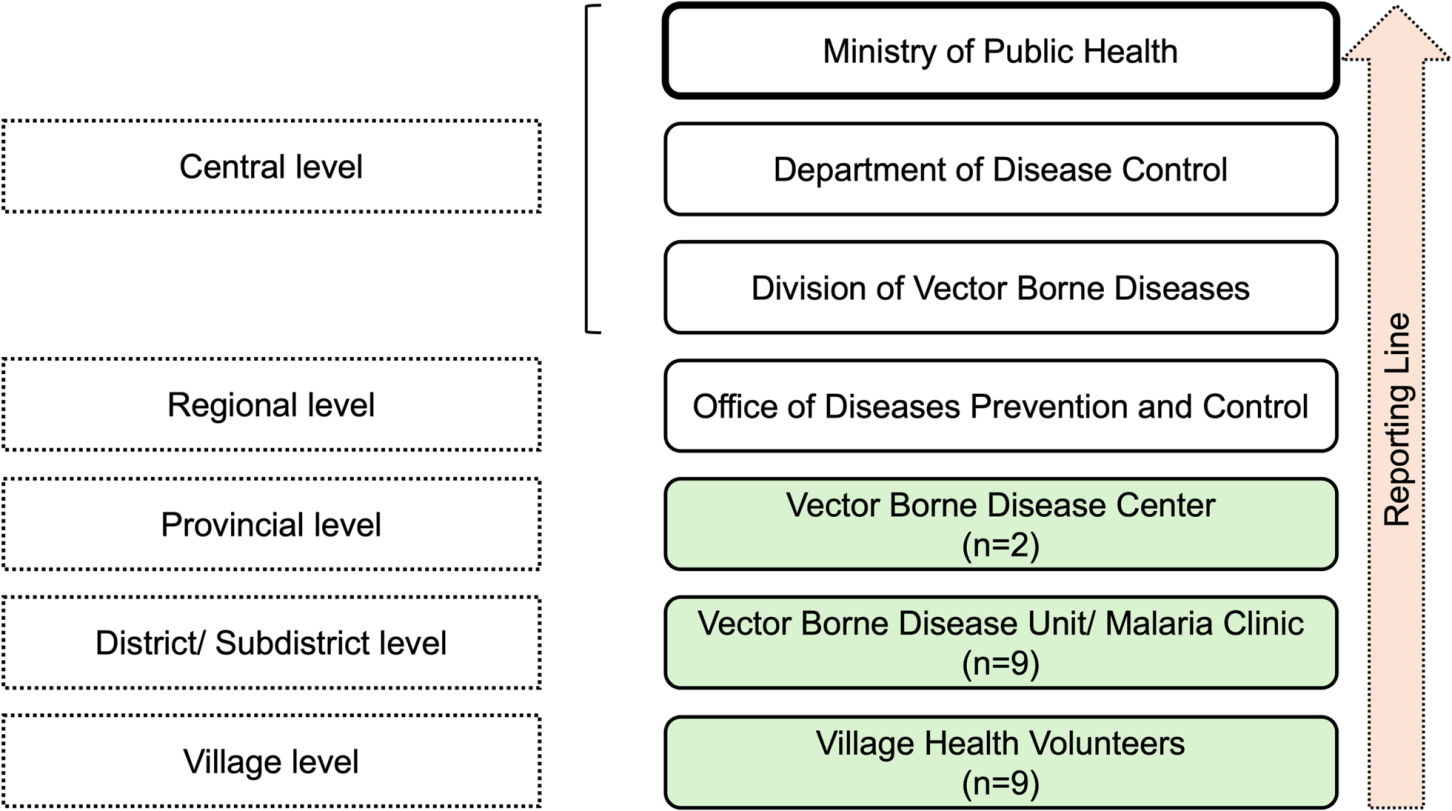
Malaria reporting flowchart (Green indicates the levels of study respondents in this study)

The selected units included the Vector Borne Disease Centre (VBDC) in Tak Province; the Vector Borne Disease Units (VBDUs) and malaria clinics (MCs) at district and sub-district levels in the three selected districts; and village health volunteers at the village level within these districts. A representative number of respondents were recruited from each level of malaria control management, ranging from two to nine individuals per level (Fig. 3). Specifically, from the VBDC overseeing all three study districts, two respondents were recruited. In each district, one VBDU or MC with the highest number of malaria cases reported was purposively selected, and approximately three respondents were recruited from each selected site, totalling nine respondents at the district/sub-district level. Under each selected VBDU/MC, three VHVs were recruited, totalling nine VHVs across the three study districts.

Eligible participants were (i) male or female, (ii) aged 18 years or older, and (iii) had a minimum of five years of experience working in malaria control in the selected areas to ensure familiarity with both historical and recent malaria trends. While gender balance was not strictly enforced, recommendations from local health staff and village leaders were considered during the selection of VHVs to ensure diversity and representation of community perspectives. In total, 20 healthcare providers and VHVs representing different levels of malaria control management from the three selected districts participated in this study (Fig. 3).

Participants were identified in collaboration with local malaria staff at the VBDC, VBDUs, and MCs. Initial contact was made through official communications. Once individuals expressed interest in participating, appointments for semi-structured interviews were scheduled at a convenient time and location, typically at their respective workplaces.

In Thailand, VHVs play a critical role in strengthening primary healthcare, particularly in rural areas, with approximately one million VHVs nationwide. Each VHV is typically responsible for health-related activities such as health education, home visits to monitor residents'health, providing basic medical services (e.g., first aid), referring suspected malaria cases for blood testing, and collecting public health data. VHVs voluntarily support these activities and receive approximately 1,000 THB per month from the MOPH as compensation for their time [27]. VHVs operate within the administrative framework of the general health services programme under the Bureau of Epidemiology, functioning parallel to the malaria programme. Positioned at village-level malaria posts, they coordinate with hospitals (of all types) and Provincial Health Offices. Despite this parallel structure, VHVs maintain close collaboration with malaria clinics to execute field-level malaria control activities, including the 1–3–7 surveillance strategy.

### Data collection

Qualitative data were collected using a semi-structured interview guide consisting of five open-ended questions, with probes to elicit deeper responses. The interview guide was developed in Thai by the research team based on a review of relevant literature, malaria trends, migration patterns, and malaria control activities in the study areas. In addition to collecting basic demographic information (e.g., age, gender, years of service), the guide focused on four pre-identified themes: (1) the current malaria situation compared to the last five years, (2) an overview of malaria control activities, (3) challenges in implementing these activities for Myanmar migrants, and (4) suggested solutions for future interventions.

To assist with data collection, two master’s level research assistants from the Mahidol Vivax Research Unit, who had substantial field experience, were given an orientation session covering the study design, qualitative interview guidelines, and ethical considerations. The data collectors visited the respondents'workplaces and conducted semi-structured interviews. While one interviewer led the questioning, the other recorded the responses in writing. Two PhD-level researchers supervised the data collection process to ensure accuracy and facilitate any necessary follow-up with the participants. Each interview was conducted in Thai and lasted approximately 30 min.

### Data entry and analysis

The collected qualitative data were first transcribed into a digital format by one research team member, followed by cross-checking for accuracy by another team member. The verbatim transcripts were then translated into English using a back-translation method to ensure consistency and accuracy. Any discrepancies encountered during the translation process were resolved by a third team member who acted as a referee. Once finalized, the data were reviewed for any language errors and subsequently imported into NVivo (Version 14) for thematic analysis [28].

A combined deductive and inductive approach was utilized for the analysis, following five sequential steps. Firstly, researchers conducted a thorough review of all transcripts and made preliminary notes to identify patterns and potential codes. Second, relevant codes were assigned to the data based on content and key points identified in the transcripts. Third, as major themes had been pre-identified prior to data collection, related codes were grouped into sub-themes. Fourth, the sub-themes were reviewed to ensure they accurately represented the data, with necessary modifications, such as rephrasing terminology or shifting themes, made to improve clarity and consistency. Finally, the themes and sub-themes were clearly defined, named, and agreed upon by all researchers to complete the thematic analysis.

## Results

In this qualitative study, a total of 20 healthcare providers and VHVs participated in semi-structured interviews. Of these, approximately 80% were from the district level or below, representing frontline staff and community-level representatives from three districts. The remaining participants were from higher administrative levels, up to the provincial level, to provide a broader or management-level perspective.

The participants had an average age of 46.9 years, ranging from 27 to 59 years. The majority (70%) were male. Most of the interviewees had extensive experience in infectious disease control, including malaria, with an average of 20 years of experience, spanning from 5 to 35 years.

### Routine malaria control and 1–3–7 surveillance implementation

Respondents demonstrated a solid understanding of ongoing malaria control activities, which were implemented consistently across the three study districts—regions with the highest malaria burden located along the Myanmar border. These activities included case diagnosis and treatment, case investigation, RCD, seasonal active case detection, health education to increase awareness and cooperation, distribution of LLINs, insecticide spraying, and weekly monitoring of unusual malaria trends in collaboration with the data team from the Division of Vector-Borne Diseases.

The “1–3–7” strategy, which integrates most malaria-related activities, was a particular focus for many respondents. This strategy entails notification of malaria cases within 24 h of confirmation, patient classification (imported vs. locally acquired/indigenous) within 72 h, and follow-up actions within a week. Follow-up measures may include RCD, indoor residual spraying (IRS), LLIN distribution, malaria testing, health education, and vector control activities around the index case's household, especially for *Plasmodium falciparum* cases. Overall, respondents estimated the operational success rates at approximately 90% for Thai residents, while the rates for migrants were significantly lower, at about 60%. Nearly all respondents cited challenges such as achieving high coverage and a low overall success rate in implementing the 1–3–7 strategy for surveillance among Myanmar migrants.

*“The 1–3–7 strategy involves blood collection, insecticide spraying within 50 households of the patient, and LLIN distribution. The operational coverage is around 90–100% for Thai residents but drops to 60–70% for migrants.”* (Healthcare provider)

*“We conduct blood surveys around the malaria patient's household, followed by IRS spraying and distribution of bed nets.”* (Village health volunteer)

*“Upon receiving case reports from hospitals, malaria posts, and clinics, we carry out a case classification survey near the index case's household or areas with high malaria incidence. We investigate the patient's travel history and then proceed with IRS spraying. In some cases, control measures are also implemented where transmission is suspected.” *(Healthcare provider)

### Increase in malaria cases compared to the last five years likely related to population movement from Myanmar

Respondents widely acknowledged that malaria control in the study areas had shown significant progress up to 2021, particularly during the COVID-19 pandemic. However, from 2022 onwards, there was a marked increase in malaria cases, including the re-emergence of *P. falciparum* in regions previously free from reported cases for several years. This resurgence was attributed to a large influx of migrants from Myanmar, driven by political turmoil, who sought employment in agriculture and frequently visited family members across borders. Many of these migrants exhibited limited knowledge of malaria prevention and poor adherence to prescribed treatments, likely due to their highly mobile lifestyles, which also restricted their participation in health campaigns.

*“In my opinion, the malaria situation nowadays has worsened compared to five years ago, mainly because of the large population migration from Myanmar following the political unrest. Many of these migrants work in agricultural areas near forests and often do not complete their prescribed treatments, resulting in repeated infections.”* (Healthcare provider)

*“Malaria cases significantly decreased during the COVID-19 period but started to rise again three years later, coinciding with increased migration from Myanmar. Many migrants lack information about malaria prevention and do not use mosquito nets or repellents.”* (Healthcare provider)

Malaria transmission was often concentrated among high-risk groups, such as short-term Myanmar migrants and those residing along the Thai-Myanmar border, rather than among long-term migrants who had lived in Thailand for at least six months. Respondents emphasized the link between migration patterns and malaria transmission. For example, migrants who travelled to Thailand for seasonal agricultural work, typically from May to December, could potentially carry and spread malaria, particularly because they often stayed in forested areas where transmission risk was higher. Similarly, some Thai nationals crossed the border to Myanmar for agricultural activities, taking advantage of lower land costs. Forest-related work, such as hunting and gathering forest products for traditional remedies, may have further increased exposure to malaria.

*“Most malaria cases involve short-term migrants. The recent surge is linked to political instability in Myanmar, leading to an influx of people crossing the border, often without proper malaria testing before starting work.”* (Village health volunteer)

*“In addition to Myanmar migrants, many Thai ethnic individuals also own land across the border for corn farming, where they stay overnight during the planting and harvest season, typically from May to December, which increases the risk of infection. The employment of temporary workers also contributes to the issue, as they often neglect protective measures.”* (Healthcare provider)

### Challenges in malaria control and surveillance among Myanmar migrants

#### Difficulties in accessing to Myanmar migrant populations

Respondents highlighted that malaria risk is exceptionally high in remote, hard-to-reach areas. Consequently, healthcare staff must travel to these locations to carry out follow-up actions to prevent onward transmission. However, this is especially challenging during the rainy season, when deteriorating road conditions make transportation difficult. Even when healthcare staff reach these communities, residents are often away during the day, working on farms far from their villages without phone signal coverage. Additionally, some people regularly cross back into Myanmar, limiting their participation in malaria control activities. Others refuse IRS in their homes due to concerns about insecticide toxicity.

*“Villagers aren't at home during the day, and traveling to reach patients is difficult during the rainy season. Mosquito nets are distributed periodically, but only about 70% of households agree to allow spraying.”* (Healthcare provider)

*“Most of the time, you won’t find villagers at home because they are working on farms.”* (Village health volunteer)

*“Addresses are often incorrect, making it difficult to locate patients. Names and identification do not match, and some individuals frequently travel to Myanmar.”* (Village health volunteer)

*“Myanmar migrants are highly mobile, making follow-up difficult.”* (Village health volunteer)

#### Language and communication barriers

Language differences pose additional challenges for reaching Myanmar migrants. Most migrants are Karen, Mon, or Burmese, but they come from diverse ethnic backgrounds and have varying levels of proficiency in Thai. Even Thai residents from remote areas may speak in a local dialect that healthcare staff find difficult to understand. This often complicates communication, necessitating the involvement of local students or community members to act as interpreters.

*“Language barriers and lack of cooperation from employers, who object to interruptions during working hours, add to the challenges of conducting surveillance among migrants.”* (Healthcare provider)

*“Communication with Myanmar migrants is challenging due to language barriers, and many lack health information.”* (Village health volunteer)

*“Some patients are Thai nationals with local dialects that healthcare staff cannot understand, especially elderly individuals, so we need translators.”* (Healthcare provider)

#### Delays in diagnosis because of limited infrastructure

Limited mobile network coverage frequently delays case notifications. Depending on the index case’s location, healthcare staff must choose between using malaria rapid diagnostic tests (RDTs) or microscopy. In areas without electricity or in situations requiring travel through rough terrain during heavy rain, RDTs are the only option. Microscopy remains the standard diagnostic method, especially for detecting low parasitaemia, and differentiating parasite stages and species other than *P. falciparum* and *P. vivax*. However, using RDTs as an initial step increases the workload since cases must later be confirmed at the clinic, potentially causing delays in diagnosis. In some instances, sample collection must be done using a flashlight or phone light due to the lack of electricity.

*“For 1–3–7 surveillance, we use either RDTs or microscopy based on whether there is access to electricity. We also use RDTs for individuals presenting with fever during surveillance.”* (Healthcare provider)

*“In the field, we use RDTs initially, followed by blood smear slides for confirmation.”* (Village health volunteer)

*“Microscopy is used in some areas, but in places without electricity, RDTs are employed first, with confirmation done later.” *(Healthcare provider)

*“Traveling to find malaria patients is challenging during the rainy season because of road conditions. Without electricity in some villages, we cannot perform microscopy, so RDTs must be used.”* (Healthcare provider)

#### Inconsistent demographic information

Respondents reported frequent discrepancies between recorded information and reality during surveillance activities. Patient names, addresses, and identification details (e.g. age and gender) often do not match official documents, especially for Myanmar migrants, who may lack passports or other official documentation. Furthermore, farm owners and households sometimes withhold information about undocumented migrants, limiting the reach of surveillance efforts. Information gathered during interviews, such as travel history, may also be unreliable.

*“The information we have often does not match reality, particularly for Myanmar migrants. Names and addresses are frequently incorrect.”* (Healthcare provider)

*“Work schedules change frequently, leading to difficulties in reaching people. Some employers and household heads hide information about short-term migrants.”* (Village health volunteer)

#### Continued migration and incomplete surveillance coverage

Political instability in Myanmar continues to drive migration into Thailand, with many individuals entering malaria-endemic areas. While some migrants eventually come under the surveillance system, newcomers often remain outside its scope. These individuals typically exhibit poor malaria prevention practices and limited healthcare-seeking behaviour, further complicating control efforts.

*“The number of Myanmar migrants crossing the border has increased due to political instability. Many enter high-risk areas with inadequate preventive measures and are not covered by the surveillance system unless they visit clinics for testing.”* (Healthcare provider)

#### Insufficient resources and logistical support

Despite efforts to estimate resources and budget for malaria control operations, unforeseen increases in malaria cases, especially in remote or forested locations, resulted in resource shortages. These included insufficient funding for transportation, limited manpower during peak case periods, and a need for additional vehicles for conducting field-based activities. The process of requesting extra budget allocations involved multiple approval steps, often leading to delays. There were also issues with the availability of essential equipment, such as RDTs, IRS sprayers, and needles suitable for small children.

“*The budget is insufficient to cover extra activities when multiple cases occur simultaneously. The approval process for additional funds can be time-consuming, and we often face manpower shortages during peak times. There is also a need for better equipment, including RDTs and child-friendly needles.”* (Healthcare provider)

*“The daily allowance is inadequate when staff have to use their own vehicles for fieldwork. In some areas, electricity is not available, making it impossible to use light microscopes for diagnosis.”* (Healthcare provider)

### Suggested solutions to improve malaria control among Myanmar migrants

#### Strengthening collaboration with local communities and organizations

Respondents recommended stronger partnerships with non-governmental health organizations (NGOs), employers of Myanmar migrants, and local community leaders. Such collaborations could improve the reporting of suspected malaria cases, facilitate malaria testing for new workers, and help maintain updated population records. Employers should also ensure that migrants undergo malaria testing when they enter high-risk areas or return from Myanmar. Community education sessions could further reinforce preventive practices.

*“Employers should notify health authorities if migrants stay longer than six months and ensure that they receive malaria-related services.”* (Healthcare provider)

*“Employers must report suspected malaria cases among workers to local authorities and malaria posts for further action.”* (Healthcare provider)

*“Employers should register and arrange malaria testing for all workers before they start work.”* (Village health volunteer)

*“Village leaders and community members should help monitor newly arrived workers who may be undocumented, ensuring they receive testing and health education.”* (Village health volunteer)

#### Expanding targeted malaria control interventions at the border

Respondents acknowledged that controlling movement across the Thai-Myanmar border is challenging, as people can easily cross rivers in remote areas without passing through official immigration channels. They suggested expanding malaria surveillance posts near border checkpoints, conducting regular mass blood surveys targeting migrants, and appointing local focal points to monitor population movements. Specific clinics or health posts could also be established along the border to provide free malaria testing and treatment. Increasing awareness about malaria testing among law enforcement agencies, border police, and the military was also recommended to help bolster control efforts.

*“Due to political instability in Myanmar, many people cross the border into Thailand, increasing the risk of malaria transmission. Limiting border crossings may help reduce transmission.”* (Village health volunteer)

*“Surveillance posts should be set up near border checkpoints specifically for Myanmar migrants.”* (Healthcare provider)

*“Establishing malaria testing facilities at the border and offering regular mass testing could help control transmission.” *(Healthcare provider)

#### Mapping and tracking migrant movements

To improve surveillance coverage, respondents suggested that employers, village leaders, and households provide healthcare staff with updated information on migrants, including personal details, travel history, and malaria status. Strengthening communication with NGOs, was also recommended to facilitate timely information sharing.

*“We need a tracking system for Myanmar migrants, especially short-term workers, including their work locations and return dates.”* (Healthcare provider)

*“Collecting detailed history from migrants, such as their travel plans, would help solve contact issues and enable follow-up appointments for blood collection.”* (Healthcare provider)

#### Improving infrastructure to support surveillance

Respondents emphasized that poor infrastructure hinders malaria control efforts. They hoped for improvements in road conditions, expanded mobile network coverage, and increased access to electricity in remote malaria-endemic areas, which would enhance operational efficiency.

*“Reaching migrants in remote areas is difficult due to poor communication and transportation infrastructure.”* (Village health volunteer)

*“Some houses lack electricity, requiring us to use flashlights or phone lights for home blood testing and follow-up.”* (Healthcare provider)

*“Communication issues arise due to the lack of phone signals and electricity, causing delays in case notifications.”* (Healthcare provider)

## Discussion

Achieving Thailand’s goal of malaria elimination by 2030 faces significant challenges, with recent increases in caseloads and border malaria being major complicating factors [16]. The recent increase in cases is likely driven by political instability in Myanmar and subsequent displacement of populations into Thailand. Implementing effective surveillance and control measures among these mobile and remote populations is difficult due to their high mobility, limited access to healthcare services, and inadequate use of preventive practices [29]. This study highlights healthcare providers' observations of an increasing malaria burden in their regions, largely attributed to cross-border transmission from Myanmar, and proposes potential solutions for future control strategies. Thailand’s malaria control efforts are constrained by the inability to implement measures across the border, underscoring the need to strengthen domestic interventions to interrupt localized transmission. This is particularly critical given the challenges of vector control in areas where major malaria vectors such as *An. dirus* and *An. maculatus* are widespread [20, 21]. Without addressing these border-related issues, malaria will likely continue to pose a significant threat to elimination efforts within the desired timeframe. Experiences from other countries show that border malaria can reintroduce the disease even in advanced stages of elimination [30–32].

Community involvement is crucial for the success of health interventions, including malaria control. Many respondents in this study highlighted the challenges posed by the transient nature of Myanmar migrants, especially short-term migrants, which hampers the coverage of malaria control activities. A significant portion of these migrants lack access to malaria services, including health education, diagnosis, treatment, and prevention [22]. Barriers such as language differences and fears of deportation due to undocumented status further limit their access to care [25, 33]. Although some health education materials are available in Thai and Karen, many migrants cannot read these languages due to limited formal education, even if they understand spoken Thai. Furthermore, there are many different regional Karen language variants and dialects, further complicating the provision of health materials in this group of languages. The challenging economic conditions and political unrest in Myanmar drive many migrants to prioritize work opportunities in Thailand over healthcare needs, including malaria prevention and treatment. Additionally, many migrants have poor knowledge, attitudes, and practices regarding malaria, making them particularly vulnerable to infection [22, 33, 34]. Consequently, relying solely on passive case detection and voluntary participation in control campaigns may not be sufficient. Instead, active approaches, such as mandatory testing policies and health education campaigns at workplaces, might be more effective, provided resources are available.

The recent resurgence of malaria cases in Thailand has been linked to the importation of infections from Myanmar migrants, underscoring the need for tailored interventions targeting this population. On the Myanmar side of the border, malaria control is limited, with most activities supported by NGOs and ethnic health organizations. Ongoing conflicts further exacerbate the malaria burden in these regions, as there is a recognized linkage between malaria and conflict [35]. Strengthening collaborations with these organizations and providing targeted support could help reduce the disease burden across the border, ultimately benefiting Thailand. Many Myanmar migrants in Thailand rely on NGOs for primary healthcare, including malaria treatment [24]. Establishing a system for timely data sharing, case classification, and coordinated follow-up actions between NGOs and local health authorities could improve malaria control outcomes. The involvement of private sector stakeholders, such as farm owners and local leaders, including VHVs, is also crucial [36]. For example, whenever a new migrant arrives, collaboration with healthcare staff to register the individual, conduct malaria testing, and provide necessary treatments and preventive measures, such as LLINs, should become standard practice. Expanding border malaria posts that specifically target migrants could also prove beneficial, as suggested by previous research [37].

Migration is closely associated with increased malaria vulnerability, especially in areas with high transmission potential. For instance, when border closures were enforced to contain the spread of COVID-19, the resulting reduction in human mobility led to a significant decrease in malaria transmission, highlighting the potential impact of migration on malaria dynamics [8, 38]. Other studies have also established the association between population movement and malaria risk [39, 40]. However, tracking all migrant mobility patterns remains challenging due to their highly mobile nature and ethical concerns, making it difficult to predict the sources of infection. Healthcare providers in this study recommended establishing policies for timely updates on new migrant arrivals, involving farm owners, company managers, and VHVs who are familiar with the local population. Such updates should be regularly maintained to track any changes in residence. These focal persons could also play a role in ensuring treatment compliance and facilitating early testing when malaria-like symptoms arise among migrants. This approach could be more cost-effective than directly observed therapy by healthcare providers, which requires intensive human resources, especially in high-transmission areas [41]. Ensuring treatment adherence is crucial, particularly in Thailand, where over 90% of reported cases are *P. vivax* infections, requiring a 14-day primaquine regimen [4, 8].

Fluctuations in malaria transmission and unexpected case surges pose challenges for estimating and budgeting the necessary commodities for malaria control. When locally acquired cases are numerous, response measures such as the 1–3–7 strategy can be labour-intensive and costly, leading to resource constraints [12]. Several respondents in this study noted periodic shortages of manpower and financial resources in their areas. Although a direct link between socio-economic development and malaria reduction is not always evident, improvements in infrastructure can indirectly support malaria control [42]. For instance, the expansion of road networks and increasing electricity access can facilitate more effective malaria diagnostics through microscopy and enable real-time case notification via mobile networks. A study showed that only 24.4% to 89.3% of malaria cases are reported within 24 h, often due to a lack of mobile network coverage. Moreover, the success of follow-up actions, such as RCD and IRS, is contingent on adequate transportation infrastructure. The same study reported an overall success rate of just 37.9% to 87.2% for responses within seven days, highlighting the need for better infrastructure to support malaria elimination efforts [13]. Therefore, considering infrastructure improvements as part of malaria control strategies may be warranted, potentially in partnership with local private companies.

This study has notable strengths and limitations. To our knowledge, it is the first to investigate healthcare provider perspectives on malaria control among Myanmar migrants in Thailand, particularly in the context of the recent political unrest in Myanmar. The use of a qualitative approach allowed for an in-depth exploration of the complexities of malaria challenges along the Thailand-Myanmar border. This approach facilitated a comprehensive understanding of the issues faced in these areas from the perspectives of healthcare providers, providing insights that may not be captured through quantitative methods alone. However, the proposed solutions derived from this study should be considered preliminary and may require adjustment based on the availability of resources; the feasibility of technical, financial, and operational factors; and ethical concerns related in part to managing healthcare systems in the midst of population displacement because of violent conflict. Additionally, the study was conducted among healthcare providers in three districts in western Thailand, which may limit the generalizability of the findings to other border regions with differing geographical characteristics, migration patterns, and malaria epidemiology. The data collection occurred in early 2024, during a period of ongoing political instability in Myanmar. Given the potential for increased migration due to evolving political circumstances, the study provides valuable baseline information for planning countermeasures to address a possible rise in malaria cases. Moreover, data were collected only up to the provincial level from village-level healthcare providers. Therefore, the findings may not fully represent the perspectives of higher-level administration. To gain a deeper understanding of the situation and challenges, future studies should also triangulate insights from Myanmar migrants residing in Thailand.

## Conclusions

Findings from this study indicate that ongoing cross-border migration from Myanmar is a key driver of malaria transmission in western Thailand, complicating efforts to achieve elimination. Healthcare providers identified persistent gaps in reaching migrant populations, ensuring timely diagnosis, and maintaining effective surveillance in remote and underserved areas. To address these challenges, targeted and migrant-responsive investments are recommended, including the deployment of mobile malaria posts near informal border crossings, engagement of trusted local actors to support follow-up and treatment adherence, and improvements in infrastructure such as electricity and network access for real-time case detection and reporting. Sustained funding will be critical to implementing these strategies and advancing malaria elimination in Thailand’s border regions. Cost-effectiveness evaluations may help prioritize interventions and support funding decisions for sustainable malaria control.

## Data Availability

All data generated or analyzed during this study are included in the article. The de-identified raw dataset is available from the corresponding authors upon reasonable request.

## Abbreviations

GMS: Greater Mekong subregion
IRS: Indoor residual spraying
LLINs: Long-lasting insecticidal nets
NGO: Non-governmental organization
RCD: Reactive case detection
RDT: Rapid diagnostic test
VHV: Village health volunteer
